# AD/ADRD AND SOCIAL SUPPORT ADVOCACY FOR BLACK NURSING HOME RESIDENTS

**DOI:** 10.1093/geroni/igae098.2141

**Published:** 2024-12-31

**Authors:** Dionne Bailey, Taylor Bucy, Tricia Skarphol, Tetyana Shippee

**Affiliations:** University of Minnesota, Minneapolis, Minnesota, United States; University of Kansas School of Medicine, Kansas City, Kansas, United States; University of Minnesota, Minneapolis, Minnesota, United States; University of Minnesota School Of Public Health, Minneapolis, Minnesota, United States

## Abstract

Black older adults represent 24% of all nursing home (NH) residents in the U.S., 20.2% of whom have Alzheimer’s disease and Alzheimer’s disease‐related dementias (AD/ADRD). Despite consuming a disproportionate share of NH services, when compared to their white counterparts, Black older adults experience worse health outcomes and are more likely to reside in lower-quality facilities, a direct byproduct of systemic racism. To cope with the stress of inadequate care delivery, many Black residents rely on external social support networks. Little research to date has explored how external social support is or can be, leveraged by Black residents for improved care delivery. Drawing on the Minority Stress Model, we applied thematic analysis to three focus group transcripts (n=22) conducted with AD/ADRD caregivers in Minnesota. We found that resident social support networks overwhelmingly adopted a “support through advocacy” mentality. Although these efforts are grounded in the care of their friends or family, advocacy behaviors often had to be extended to additional residents because they identified themselves as lacking social support. Given the disproportionate share of Black residents with AD/ADRD living in institutional-based settings, understanding both formal and informal care delivery practices, especially the role of Black resilience, is essential to creating high-quality care settings.

